# Proximity proteomics of C9orf72 dipeptide repeat proteins identifies molecular chaperones as modifiers of poly-GA aggregation

**DOI:** 10.1186/s40478-022-01322-x

**Published:** 2022-02-14

**Authors:** Feilin Liu, Dmytro Morderer, Melissa C. Wren, Sara A. Vettleson-Trutza, Yanzhe Wang, Benjamin E. Rabichow, Michelle R. Salemi, Brett S. Phinney, Björn Oskarsson, Dennis W. Dickson, Wilfried Rossoll

**Affiliations:** 1 Department of Neuroscience, Mayo Clinic, Jacksonville, FL USA; 2 Department of Ophthalmology, The Second Hospital of Jilin University, Changchun, China; 3 Department of Neurology, The First Affiliated Hospital of China Medical University, Shenyang, Liaoning China; 4 Proteomics Core, University of California Davis, Davis, CA USA; 5 Department of Neurology, Mayo Clinic, Jacksonville, FL USA

**Keywords:** C9orf72, Poly-GA, Proximity proteomics, Heat shock proteins

## Abstract

The most common inherited cause of two genetically and clinico-pathologically overlapping neurodegenerative diseases, amyotrophic lateral sclerosis (ALS) and frontotemporal dementia (FTD), is the presence of expanded GGGGCC intronic hexanucleotide repeats in the *C9orf72* gene. Aside from haploinsufficiency and toxic RNA foci, another non-exclusive disease mechanism is the non-canonical translation of the repeat RNA into five different dipeptide repeat proteins (DPRs), which form neuronal inclusions in affected patient brains. While evidence from cellular and animal models supports a toxic gain-of-function of pathologic poly-GA, poly-GR, and poly-PR aggregates in promoting deposition of TDP-43 pathology and neurodegeneration in affected brain areas, the relative contribution of DPRs to the disease process in c9FTD/ALS patients remains unclear. Here we have used the proximity-dependent biotin identification (BioID) proximity proteomics approach to investigate the formation and collective composition of DPR aggregates using cellular models. While interactomes of arginine rich poly-GR and poly-PR aggregates overlapped and were enriched for nucleolar and ribosomal proteins, poly-GA aggregates demonstrated a distinct association with proteasomal components, molecular chaperones (HSPA1A/HSP70, HSPA8/HSC70, VCP/p97), co-chaperones (BAG3, DNAJA1A) and other factors that regulate protein folding and degradation (SQSTM1/p62, CALR, CHIP/STUB1). Experiments in cellular models of poly-GA pathology show that molecular chaperones and co-chaperones are sequestered to the periphery of dense cytoplasmic aggregates, causing depletion from their typical cellular localization. Their involvement in the pathologic process is confirmed in autopsy brain tissue, where HSPA8, BAG3, VCP, and its adapter protein UBXN6 show a close association with poly-GA aggregates in the frontal cortex, temporal cortex, and hippocampus of c9FTLD and c9ALS cases. The association of heat shock proteins and co-chaperones with poly-GA led us to investigate their potential role in reducing its aggregation. We identified HSP40 co-chaperones of the DNAJB family as potent modifiers that increased the solubility of poly-GA, highlighting a possible novel therapeutic avenue and a central role of molecular chaperones in the pathogenesis of human *C9orf72*-linked diseases.

**Supplementary Information:**

The online version contains supplementary material available at 10.1186/s40478-022-01322-x.

## Introduction

Amyotrophic Lateral Sclerosis (ALS) and frontotemporal dementia (FTD) are progressive neurodegenerative diseases with overlapping clinical, genetic, and neuropathological features that represent the extreme ends of a common FTD/ALS disease spectrum with likely shared pathomechanisms [53, 70, 91]. The most common genetic cause of familial ALS and FTD is a GGGGCC hexanucleotide repeat expansion (HRE) in intron 1 of the *C9orf72* gene locus [18, 77]. Work from several labs across various model organisms have identified three different but non-exclusive disease mechanisms that may contribute to the pathophysiology in human patients [24]. First, HREs may reduce the expression of the C9orf72 protein, a guanine nucleotide exchange factor (GEF), which plays a role in autophagy [48]. Second, both the sense and antisense sequence forms RNA foci in patients and disease models that sequester cellular proteins, causing nucleocytoplasmic transport defects [16, 62]. Third, repeat RNA can be translated into all 6 reading frames via repeat associated non-ATG (RAN) translation [106]. The resulting dipeptide repeat (DPR) proteins, poly-GA, -GR, -PR, -GP, and -AP form aggregates that can be detected in the brains of FTD/ALS patients with *C9orf72* repeat expansions (c9FTD/ALS) [3, 58, 66]. Studies in cellular and animal models have found significant toxicity for the positively charged arginine-rich poly-GR and poly-PR proteins, and for the highly insoluble poly-GA aggregates [21, 86]. While poly-GP can serve as a pharmacodynamic biomarker for therapy development [23] and targeting poly-GA was found to be therapeutic in C9orf72 disease models [103], the pathomechanisms and relative contribution of DPR toxicity to human disease are still unclear. This raises important questions about (1) what proteins are sequestered into DPR aggregates, thereby causing cellular defects, and (2) what molecular pathways can modulate the formation and disassembly of DPR aggregates, as potential novel targets for therapeutic intervention.

Previous DPR interactome studies, based on co-immunoprecipitation and proteomic analysis of soluble DPR proteins, have primarily found an association between arginine-rich poly-GR and poly-PR with ribosomal proteins and RNA-binding proteins with low complexity domains (LCDs) [6, 54, 56, 98]. This suggests impairments in ribosome biogenesis and protein translation, as well as defects in the assembly and function of membraneless organelles such as stress granules and nucleoli [32, 39, 49, 64]. Poly-GA aggregates have been less well biochemically characterized, due to their highly detergent-insoluble nature, yet some studies indicate enrichment with proteasome components and other proteins involved in the ubiquitin proteasome system (UPS) [60], mediating impaired protein degradation and induction of endoplasmic reticulum (ER) stress pathways [102]. Despite these earlier studies, the contribution of poly-GA-induced pathology to the pathogenesis of c9FTD/ALS and the nature of molecular mechanisms and cellular pathways targeting these pathological aggregates for protein degradation remain poorly understood.

In this study, we employed the proximity-dependent biotin identification (BioID) approach that allows for the labeling of proximal proteins in living cells and their purification under harsh denaturing conditions [12, 80], to determine the interactome of detergent-insoluble poly-GR, -PR, and -GA aggregates. While the interactome for the arginine-rich proteins overlapped and contained primarily nucleolar and ribosomal proteins, the highly insoluble poly-GA interactome was distinct and enriched not only for proteasome components as reported previously [26, 60, 101], but we also discovered an association with numerous molecular chaperones and other proteins involved in protein folding and protein degradation pathways. Sequestration of these proteins to poly-GA aggregates in cell culture and human autopsy brain tissue suggests downstream defects in specific protein quality control pathways, which may ultimately contribute to the resulting neurodegeneration observed in disease models and human patients. Poly-GA associated protein candidates were extensively validated in both cell culture experiments and human autopsy brain tissue, showing very similar patterns of association with insoluble poly-GA aggregates. In addition, we established that the expression of specific DNAJB family co-chaperones reduced the formation of poly-GA aggregates, similar to their reported activity towards polyglutamine (poly-Q) and α-synuclein aggregates, therefore expanding their potential value as therapeutic targets for c9FTD/ALS patients.

## Materials and methods

### Cell culture and plasmid transfections

HEK293T cells (ATCC CRL-3216) were cultured in DMEM (Gibco) supplemented with 10% FBS (GenClone) and 1% Pen Strep (Thermo Fisher Scientific) and grown at 37 °C with 5% CO_2_. Cells were transfected using Lipofectamine LTX reagent (Invitrogen) following the manufacturer’s instructions.

### DNA constructs

To generate myc-BioID-DPR_x100_ plasmids, sequences encoding poly-GR, -PR, and -GA (with alternating codons to avoid RNA repeats) were cloned into the pcDNA3.1 mycBioID plasmid (Addgene plasmid #35700) [80] by replacing the TDP-CTF BamHI/HindIII fragment in pcDNA3.1 mycBioID TDP-CTF [12]. Resulting plasmids encode myc-BioID-(GGGS)_x3_-(DPR)_x100_ fusion proteins. EGFP and mCherry-tagged DPR _x100_ plasmids were generated by replacing the BioID NdeI/BamHI fragment in myc-BioID-(GGGS)_x3_-(DPR)_x100_ with EGFP or mCherry. 2xHA-poly-GA was cloned by replacing the PvuI/BamHI fragment of EGFP in EGFP-poly-GA with a double HA tag (MGYPYDVPDYAGGYPYDVPDYA). Expression constructs for EGFP-tagged SQSTM1, UBXN6, VCP, and HSPA8 (accession numbers #38277 [35], #86464 [75], # 23971 [55], #19487 [30], respectively), as well as mCherry-Calreticulin-N-16 (#55006) were obtained from Addgene. EGFP-tagged Calreticulin was cloned by replacing mCherry AgeI/NotI fragment in mCherry-Calreticulin-N-16 with EGFP from the pEGFP-N1 vector (Clontech).

### BioID labeling, affinity purification, and immunoblotting

HEK293T cells (ATCC CRL-11268) were transfected with expression plasmids for EGFP, myc-BioID-DPR_x100,_ or myc-BioID using the Lipofectamine LTX transfection reagent (Invitrogen). Biotin was added to the medium to a final concentration of 50 μM the next day. After 24 h, cells were washed three times with ice-cold PBS and lysed in denaturing urea lysis buffer (8 M urea, 50 mM Tris–HCl pH 7.5) for 20 min at room temperature (RT). Cell lysates were sonicated and centrifuged at 21,000×*g* for 20 min. For the pulldown of biotinylated proteins, Streptavidin Sepharose High Performance beads (GE Healthcare, 17511301) were incubated with the lysate sample with constant rotation overnight at 4 °C. Beads were collected by centrifugation and washed three times with urea lysis buffer and three times with 50 mM ammonium bicarbonate. Samples were divided where 15% were used for immunoblotting and silver staining, while the remaining 85% was incubated with 500 ng of sequencing-grade trypsin (Promega, V5111) in ammonium bicarbonate overnight. Beads were spun down and the supernatant was collected and dried using a SpeedVac for LC–MS/MS (see below).

For immunoblotting, samples in Bolt LDS sample buffer were separated in a Bolt 4–12% Bis–Tris polyacrylamide gradient gel (Invitrogen) and proteins were transferred to nitrocellulose membranes with the iBlot 2 Dry Blotting System (Thermo Fisher Scientific). Membranes were blocked with Intercept Blocking Buffer (LI-COR, 92770001) in phosphate-buffered saline (PBS) for 45 min, followed by incubation with anti-β-tubulin primary antibody (1:5000, DHSB, E7) overnight at 4 °C, and Alexa Fluor Plus 800-conjugated secondary antibodies (1:10,000, Invitrogen, A32730) in Intercept Blocking Buffer for 1 h at RT. Biotinylated proteins were probed with IRDye 680RD streptavidin (1:10,000, LI-COR, 92668031) and detected on an Odyssey CLx scanner (LI-COR). For silver staining, protein gels were processed with Pierce Silver Stain Kit (Thermo Fisher Scientific, 24612) according to the manufacturer’s instructions.

### Liquid chromatography tandem mass spectrometry (LC–MS/MS)

Peptides were resuspended in 2% acetonitrile, 0.1% trifluoroacetic acid, and directly loaded on a 75 µm × 10 cm, 1.9 µm particle size, C18 column (Bruker, Bremen, Germany) with Captive Spray emitter. Peptides were separated using a Bruker Nano-elute nUPLC at 500 nL/min. Solvent A = 0.1% formic acid, Solvent B = 100% Acetonitrile 0.1% formic acid. Gradient conditions = 2%B to 35%B over 25 min using a Bruker timsTOF Pro mass spectrometer. Mass spectrometry data was acquired using the diaPASEF PY9 method [63]. The acquisition scheme used for diaPASEF consisted of four 25 *m*/*z* precursor windows per 100 ms TIMS scan. Sixteen TIMS scans, creating 64 total windows, layered the double and triple charged peptides on the *m*/*z* and ion mobility plane. Precursor windows began at 400 *m*/*z* and continued to 1200 *m*/*z*. The collision energy was ramped linearly as a function of the mobility from 63 eV at 1/K0 = 1.5 Vs cm^−2^ to 17 eV at 1/K0 = 0.55 Vs cm^−2^. Some of the samples have been run twice as technical replicates.

### Proteomic data analysis

Data-independent acquisition (DIA) data was analyzed with the Spectronaut 14 software package (Biognosys) using the DIA targeted library-based search. Libraries were generated by searching the diaPASEF data using the Spectronaut pulsar search engine with the default settings against Uniprot UP000005640 Human (78,120 entries) supplemented with the myc-BioID protein sequence. DiaPASEF data were matched against the resulting libraries using Spectronaut 14 default settings. Briefly, “trypsin/P specific” was set for the enzyme allowing two missed cleavages, fixed modifications were set to cysteine carbamidomethylation, and variable modification were set to peptide N-terminal acetylation and methionine oxidation. For DIA search identification, PSM and Protein Group decoy false discovery was set at 1%. Summarization of protein intensities and statistical comparison of protein abundances between the studied groups was performed with the MSstats R package (version 4.0.1) [11] using R (version 4.1.1.) and Rstudio (version 1.4.1717). Proteins were considered as specific interactors when: (1) the log2 fold-changes of protein abundances in the corresponding samples comparing to both myc-BioID and EGFP controls were > 1; (2) the Benjamini–Hochberg adjusted *p* values in both comparisons were < 0.05; and (3) there were no more than 50% missing values for the protein in the corresponding bait and myc-BioID control samples. Analysis for the enrichment of Gene Ontology (GO) terms and Kyoto encyclopedia of genes and genomes (KEGG) pathways in the obtained protein datasets was performed with the g:Profiler web-based tool using “Only annotated genes” as the statistical domain scope and Benjamini–Hochberg FDR < 0.05 as the significance threshold [76]. Plotting was performed using ggplot2 (version 3.3.5) [95], ggfortify (version 0.4.12) [92], ggrepel (version 0.9.1), RColorBrewer (version 1.1–2) and heatmap (version 1.0.12) R packages. Protein interaction networks were imported from the STRING database [90] using the interaction combined cutoff score of 0.7 and visualized using the Cytoscape software platform (version 3.8.2) [87]. Venn diagrams were generated using the BioVenn online tool [34].

### Immunofluorescence, image acquisition, and analysis in cell culture

HEK293T cells were grown on poly-l-lysine–coated coverslips overnight and co-transfected with expression plasmids for EGFP or epitope-tagged protein of interest and mCherry or mCherry-tagged-poly GA. Cells were incubated with primary antibodies including anti-myc tag (1:50, DSHB, 9E10), anti-HA tag(1:1000, BioLegend, 901514), anti-BAG3 (1:200, Proteintech, 10599-1-AP), anti-SQSTM1 (1:500, Abcam, ab91526), anti-UBXN6 (1:100, Proteintech, 14706-1-AP), anti-VCP (1:500, Abcam, ab109240), anti-V5 tag (1:500, Invitrogen, R960-25), overnight at 4 °C. After washing in PBS, the coverslips were incubated with Alexa Fluor 488- or 555-conjugated secondary anti-rabbit or anti-mouse antibodies (1:1000, Invitrogen, A32723, A32727, A32731 or A32732) and, where applicable, with DyLight 650-conjugated neutravidin (1:500, Invitrogen, 84607), for 1 h at RT. Nuclei were counterstained with Hoechst 33342 (1:5000, Thermo Fisher Scientific, 62249) and the coverslips were mounted on glass slides using ProLong Glass Antifade Mountant (Invitrogen, P36980).

For high-resolution imaging, z-series were acquired according to the different experimental designs with an ECLIPSE Ti2 fluorescence microscope (Nikon) equipped with a Spectra X multi-LED light engine (Lumencor), single bandpass filter cubes for DAPI, EGFP/FITC, mCherry, and Cy5/AF647 (Chroma), and a ZYLA 4.2 PLUS sCMOS camera (Andor), using NIS Elements HC V5.30 software (Nikon). Within each experiment, all groups were imaged with the same acquisition settings. Imaging parameters were set so that the obtained pixel fluorescence intensity was within the dynamic range of the camera to avoid overexposure. Out-of-focus blur was removed from z-series of fluorescence images via three-dimensional (3D) deconvolution with the NIS-Elements Advanced Research (V5.30) deconvolution package.

### Co-immunofluorescence on human post-mortem tissue

All post-mortem cases were provided by the Mayo Clinic Florida Brain Bank. Tissue was obtained from patients who were carriers of the *C9orf72* repeat expansion and had a confirmed neuropathological diagnosis of either ALS or frontotemporal lobar degeneration (FTLD). Information on the post-mortem cases is provided in Table 1. Informed written consent was obtained before brain donation and study entry from all patients or their legal next of kin when required. Biological samples were obtained with Mayo Clinic Institutional Review Board approval. Donated brains were fixed in 10% neutral buffered formalin before blocks were sectioned and embedded in paraffin for their preservation and sectioning. Double immunofluorescence staining was performed on 5 µm formalin fixed paraffin embedded (FFPE) tissue sections obtained from the mid frontal cortex, superior temporal cortex and medial temporal lobe containing anterior hippocampus. In brief, FFPE tissue sections from the respective post-mortem human brain areas were deparaffinized by immersion in several successions of xylene and rehydrated through a series of graded ethanol solutions (100%, 90% and 70% ethanol), before rinsing in dH_2_O and equilibration in Tris-buffered saline (TBS). Antigen retrieval was performed by steaming tissue sections in citrate buffer, pH 6 (Dako Target retrieval solution, S2369) for 30 min. Slides were slowly cooled for 15 min and gently rinsed with dH_2_O for 10 min. Sections were permeabilized with TBS containing 0.3% Triton-X 100 at RT for 15 min, washed three times for 5 min with TBS, and subsequently blocked with serum-free protein block (Dako, X0909) for 1 h at RT. Tissues were immunostained with antibodies against poly-GA (1:1000, EMD Millipore, MABN889) together with anti-SQSTM1 (1:200, Abcam, ab91526), anti-HSPA8/HSC70 (1:200, Proteintech, 10654-1-AP), anti-VCP (1:200, Abcam, ab109240), anti-UBXN6 (1:200, Proteintech, 14706-1AP) or anti-BAG3 (1:200, Proteintech 10599-1-AP), which were diluted in antibody diluent (Dako, S0809) for overnight incubation at 4 °C in a humidified chamber. The following day sections were washed in TBS containing 0.05% Tween-20 (TBS-T), three times for 10 min each, and incubated with Alexa Fluor conjugated secondary antibodies (1:500, anti-mouse 488 (A32723) and anti-rabbit 555 (A32732), both from Invitrogen), diluted in antibody diluent (Dako, S0809), for 2 h at RT. Sections were washed with TBS-T, three times for 10 min each, and then incubated with Hoechst 33342 (1:1000, Thermo Fisher Scientific, 62249) for 20 min at RT and washed with TBS (twice, for 10 min). Lastly, tissue sections were stained with 1 × True Black lipofuscin autofluorescence quencher (Biotium, 23007) for 30 s and rinsed with dH_2_O for 5 min. Tissue sections were mounted with glass slides using ProLong Glass Antifade Mountant (Invitrogen, P36980) and imaged on an ECLIPSE Ti2 fluorescence microscope (Nikon) as described above, except out-of-focus blur was removed from z-series of fluorescence images via the Clarify.ai module of the NIS-Elements Advanced Research (V5.30) deconvolution package. Great care was taken in selecting imaging parameters to avoid overexposures. In addition to single optical sections for all channels, z-series are also shown as 3D volume rendered views to demonstrate co-localization within cells in 3D.

**Table 1:** Demographical data of human post-mortem cases used for co-immunofluorescence validation

Case ID	Clinical Dx	Pathol. Dx	Age	Sex	Genetics	TDP-43 subtype	Braak stage	Thal phase	Brain weight (g)	Disease duration
C9/ALS #1	ALS	ALS	67	M	C9orf72	B	II	0	1240	6
C9/ALS #2	ALS/MCI	ALS	67	F	C9orf72	B	II	0	1080	3
C9/FTD #1	FTD	FTLD	62	M	C9orf72	B	II	0	1060	4

ALS, amyotrophic lateral sclerosis; MCI, mild cognitive impairment; FTD, frontotemporal dementia; FTLD, frontotemporal lobar degeneration; *C9orf72*, chromosome 9 open reading frame 72; TDP-43, transactive response DNA-binding protein 43; Dx, diagnosis; M, male; F, female

### Filter trap assay (FTA)

Filter trap assays were performed essentially as described [96]. Briefly, HEK293T cells were co-transfected with EGFP-tagged poly-GA and either V5-tagged chaperones or the mCherry control plasmid as described above. After a 24 h period of transfection, cells were lysed on ice in Triton X-100 lysis buffer (1% Triton X-100, 15 mM MgCl_2_ in PBS), supplemented with Pierce Protease Inhibitor cocktail (Thermo Fisher Scientific, A32965) and 30 units of DNase I. Equal amounts of lysates were centrifuged at 21,000×*g* at 4 °C for 30 min and insoluble pellets were resuspended in SDS lysis buffer (2% SDS in 100 mM Tris, pH 7.5) for 2 h at RT. Samples were diluted at 1:5 in SDS lysis buffer and filtered through a cellulose acetate membrane (0.2 μm pore size) using the Bio-Dot SF Microfiltration System (BioRad). The membrane was blocked in Intercept Blocking Buffer for 1 h and the protein bands were detected with antibodies against GFP (1:1000, Takara Bio, 632592), followed by Alexa Fluor Plus 800-conjugated secondary antibody (1:10,000, Invitrogen, A32735), and scanned on an Odyssey CLx imaging system (LI-COR).

### Statistical analysis

Quantitative data is presented as mean ± SD. Statistical comparisons between multiple experimental groups were performed using Kruskal–Wallis test followed by post hoc pairwise comparison between control and experimental groups via a Dunn’s multiple comparison test. Statistical analysis was performed using GraphPad Prism software (V 9.2.0).

## Results

### Proximity-dependent biotin identification (BioID) of DPR-associated proteomes in HEK293T cells

**Fig. 1:**
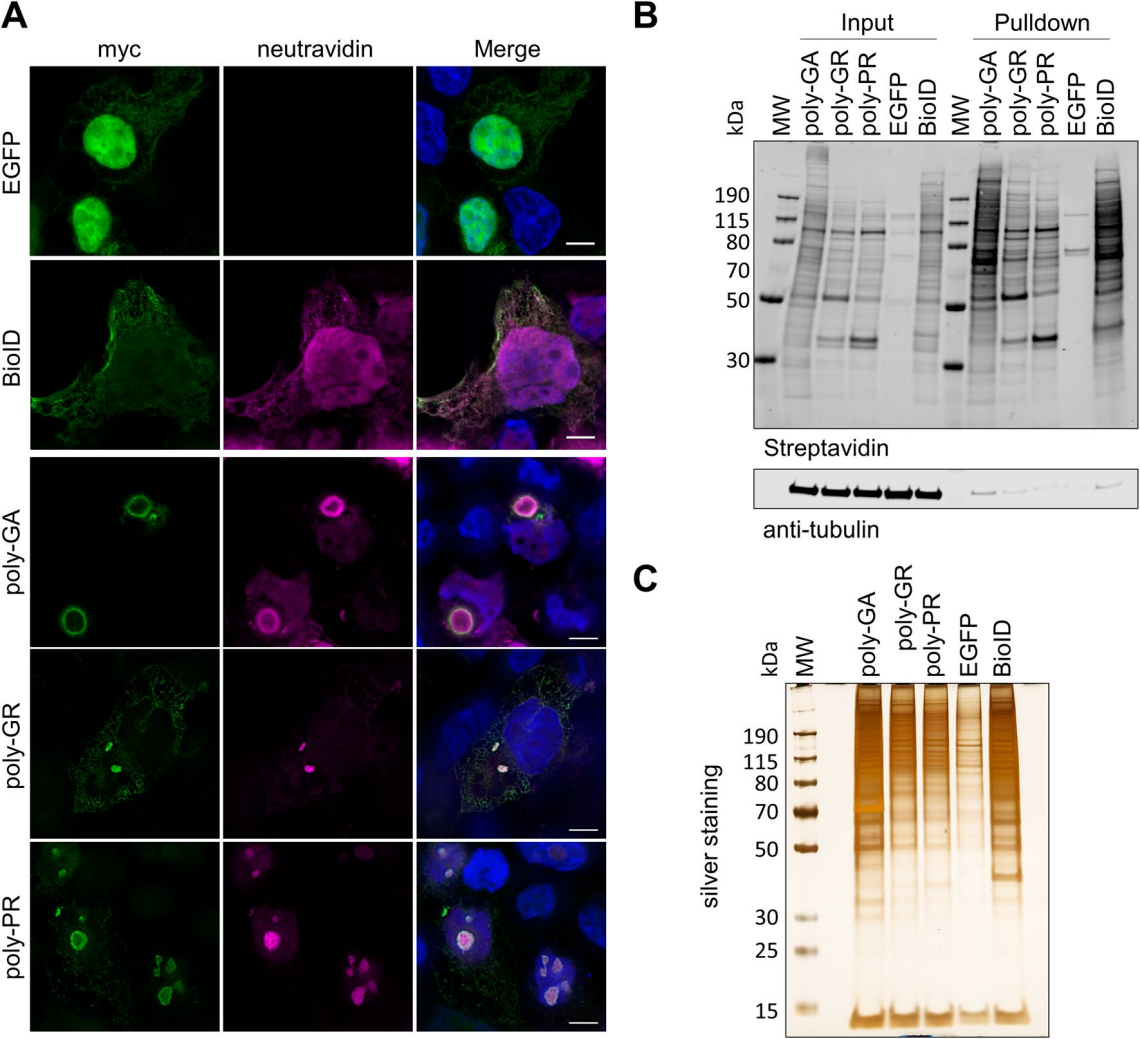
BioID of poly-GA, -GR, and -PR shows specific proximity labeling of DPR protein aggregates. **A** myc-BioID-tagged poly-GA, -GR, and -PR fusion proteins expressed in HEK293T cells form distinct aggregates (anti-myc; green) that are strongly labeled with biotin (neutravidin; magenta). EGFP-transfected cells were used as a negative control, while the myc-BioID expressing specificity control shows general nucleocytoplasmic labeling (Scale bar = 5 µm). **B** Affinity purification experiments with streptavidin beads show the enrichment of distinct biotinylated proteins in the pull-down fraction. Anti-β-tubulin was used as a loading control. **C** Silver staining shows affinity purified biotinylated proteins with low levels of endogenously biotinylated and unspecifically binding proteins in the negative control (EGFP)

The BioID assay is based on the expression of a promiscuous biotin ligase fusion protein that catalyzes the biotinylation of proximate lysine residues, to allow for the identification of proximal and interacting proteins in the context of living mammalian cells [80]. Poly-GA, poly-GR, and poly-PR encoding sequences (as 100 repeats) were generated via DNA synthesis and cloned as a C-terminal fusion connected via a flexible linker (GGGSGGGSGGGS) into a myc-BirA* plasmid encoding the promiscuous R118G mutant of the *E. coli* biotin ligase BirA (Fig. 1A)[80]. We selected the original BirA* biotin ligase with slow labeling kinetics, now also commonly referred to as BioID, over more processive variants such as TurboID that were developed to efficiently label proteins within cellular components [9], since we aimed to preferentially label protein components associated with insoluble aggregates instead of soluble proteins. To validate the approach via immunocytochemistry, HEK293T cells were transfected with BioID-DPR_x100_ expression and control constructs and incubated for 24 h to allow for the formation of protein aggregates. After incubation with 50 µM biotin to induce biotinylation of proximate proteins, immunostaining with anti-myc antibodies and fluorophore-labeled neutravidin was performed. Myc-BioID-poly-GA formed dense cytoplasmic aggregates consistent with human pathological findings, whereas myc-BioID-poly-GR was observed in both the nucleoli and cytoplasm, and myc-BioID-poly-PR was mainly nucleolar (Fig. 1A). While nuclear and para-nucleolar DPR inclusions are rare in human autopsy brain tissue, these localization patterns are consistent with previous in vitro studies on poly-GR and -PR aggregates using different tags and cell types [85]. These localization patterns suggests that BioID-fusions do not affect the localization of DPRs in cell culture. As a specificity control, we expressed myc-BioID, which showed the expected general nucleocytoplasmic distribution, while the EGFP expression plasmid was used as a negative control. Neutravidin staining demonstrates the colocalization of biotinylated proteins with myc-BioID-DPR_x100_ and myc-BioID proteins, whereas cells transfected with EGFP were negative for biotinylation.

To affinity-purify proximity-biotinylated proteins, cells were incubated with biotin as described above, washed thoroughly in PBS and lysed under harsh denaturing conditions in 8 M urea buffer to solubilize protein aggregates, as previously established for TDP-43 aggregates [12]. Western blots with fluorescent neutravidin showed distinct patterns of proximity-dependent biotinylation in myc-BioID-DPR cell lysates and pull-down samples, as compared to low abundant endogenously biotinylated proteins present in the EGFP-transfected negative control (Fig. 1B). Silver staining was used to confirm selective enrichment of purified biotinylated proteins over nonspecifically binding proteins in the negative control (Fig. 1C).

**Fig. 2:**
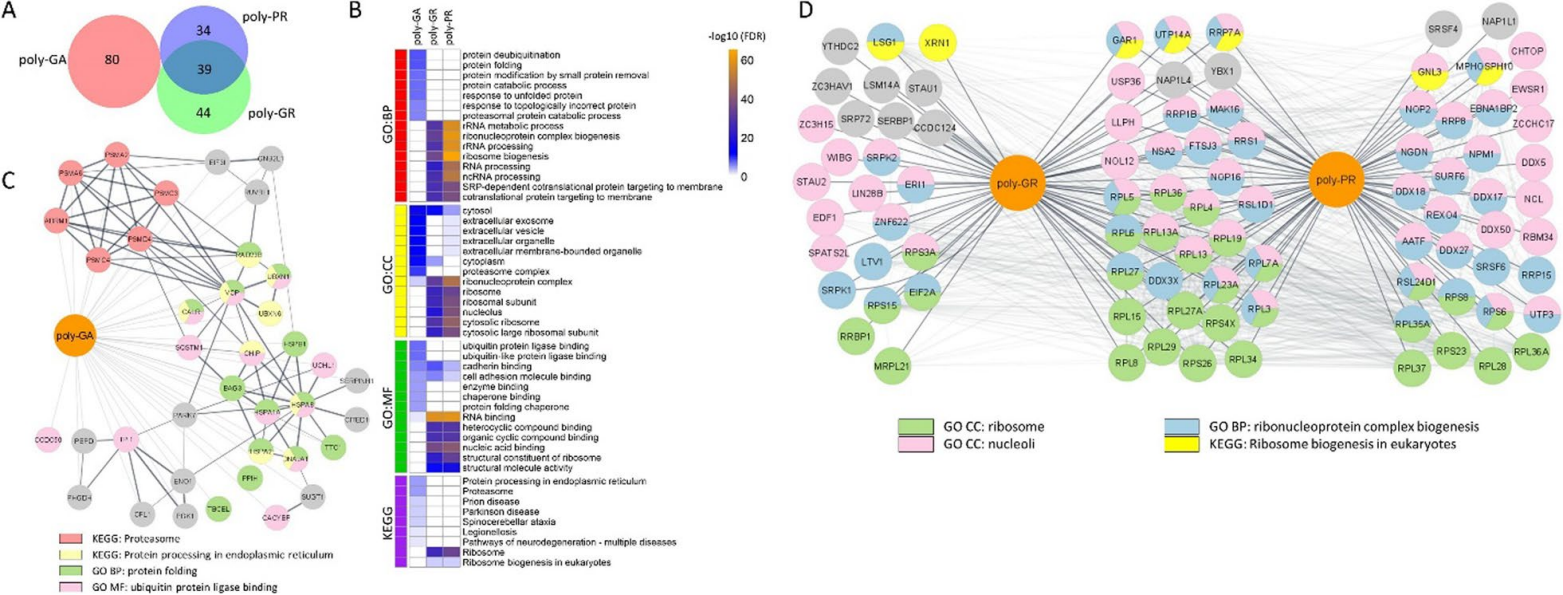
The poly-GA associated proteome is distinct from those for poly-GR and -PR. **A** Venn Diagram showing the overlap between the identified associated proteins for poly-GA, poly-GR and poly-PR dipeptide repeats; **B** Heatmap of the most enriched GO and KEGG terms in DPR-associated protein sets. Cell colors represent − log10 of the adjusted *p*-value of enrichment; **C** STRING interaction network for a subset of the identified poly-GA associated proteins that belong to the indicated GO or KEGG terms or directly interact with proteins belonging to these terms. Node colors indicate protein affiliation with the indicated terms. Solid edges represent the interactions from the STRING database, transparent edges illustrate the association between poly-GA and proteins shown here. Edge thickness corresponds to the STRING combined score of an interaction. **D** STRING interaction network for a subset of the identified poly-GR and poly-PR associated proteins that belong to the indicated GO or KEGG terms or directly interact with proteins belonging to these terms. Node colors indicate protein affiliation with the indicated terms. Transparent edges represent the interactions from the STRING database, solid edges illustrate the association between poly-GR or poly-PR and proteins shown here

For proteomic analysis of DPR-associated biotinylated proteins, streptavidin bead-bound proteins were digested with trypsin, and the obtained peptides were analyzed by data independent acquisition mass spectrometry (DIA LC–MS/MS). For each of the studied constructs or controls three biological replicates were prepared. Principal component analysis of the samples highlights tight clustering of the proximity proteomes of arginine-containing DPRs and poly-GA, that were well separated along the PC1 axis (Additional file 1: Supplementary Fig. 1A). Principal component 2 separated poly-GR from poly-PR and poly-GA from myc-BioID negative controls (Additional file 1: Supplementary Fig. 1A). By applying the stringent criteria discussed in the methods section, we have identified 80 proteins associated with poly-GA, 83 proteins associated with poly-GR and 73 proteins associated with poly-PR (Fig. 2A, Additional file 2: Supplementary Table 1). Notably, no common interactors for the arginine-rich DPRs vs. poly-GA aggregates were identified in our analysis (Fig. 2A).

### Poly-GR and poly-PR proximity proteomes overlap and are highly enriched for ribosomal and nucleolar proteins

Analysis of the GO terms and KEGG pathway enrichment was performed in each of the obtained sets of DPR interactors, showing distinct functional classifications for the poly-GA interactome vs. the strongly overlapping arginine-rich DPRs (Fig. 2B). For both poly-GR and poly-PR associated proteins the most enriched GO terms were ribosomal function or biogenesis (Fig. 2B, D). Proteins assigned to this category include ribosomal proteins, ribosome biogenesis factors, and other nucleoli resident proteins. Other proteins associated with poly-GR and poly-PR include RNA-binding proteins associated with membrane-less organelles that not only include nucleoli but also stress granules. These findings are in good agreement with the observed cellular localization of these DPRs, as well as with previous studies that reported the association of poly-GR and poly-PR with ribosomal and nucleolar proteins using different experimental models and paradigms [32, 46, 49, 64]. While many of these associated proteins were shared between poly-GR and poly-PR (i.e. RPL4, RPL5, RPL6 etc.), others were exclusively found in poly-GR (i.e. LTV1, STAU2 etc.) or poly-PR (i.e. NPM1, EWSR1, etc.) associated proteomes (Fig. 2D; Additional file 1: Supplementary Fig. 1C and D, Additional file 2: Supplementary Table 1). Several of these proteins identified in our proximity proteomics study exhibited a potential functional connection to C9orf72 pathological features. The *Drosophila* ortholog of double-stranded RNA-binding protein Staufen homolog 2 (STAU2) shows nuclear accumulation when C9orf72-associated arginine-rich dipeptide repeats are expressed, as an important pathological feature in neurons [44]. Nucleophosmin (NPM1) has been shown to disperse from nucleoli and disrupt its role in organizing ribosomal proteins and RNAs within the nucleolus upon poly-PR expression, suggesting a direct mechanistic link to protein synthesis defects in c9FTD/ALS models [94]. Rare genetic variants of the RNA-binding protein EWS (EWSR1) in ALS patients cause the formation of cytoplasmic aggregates and this protein has also been observed to co-aggregate in FTLD cases with FUS pathology, suggesting a potential role in the FTD/ALS disease process [15, 69].

### Poly-GA associates with a network of heat shock proteins and other chaperones and co-chaperones

Comparison of the poly-GA associated proteins identified in this study with previously published datasets [8, 60, 64] shows some limited overlap but also identifies 71 novel poly-GA specific interactors, probably due to different experimental approaches used (Additional file 1: Supplementary Fig. 2). The fact that we found no overlap with poly-GR and -PR associated proteins reflects the fundamental differences in the biophysical properties of these compact aggregates formed by small, aliphatic, and uncharged amino acids [21]. In contrast to the association of the poly-GR and poly-PR interactomes with the KEGG pathway terms “ribosome” and “ribosome biogenesis”, the poly-GA-associated protein set was enriched for the KEGG pathway term “proteasome”, “protein processing in endoplasmic reticulum” as well as neurodegenerative disease pathways including “Parkinson’s disease” and “spinocerebellar ataxia” that are characterized by detergent-insoluble α-synuclein or prevalent polyglutamine (poly-Q) aggregates, respectively (Fig. 2B). The identified proteasomal proteins PSMA2, PSMA6, PSMC3, PSMC4 and PSMD4, as well as proteasomal ubiquitin receptor ADRM1 (Fig. 2C), are in line with cryo-electron tomography studies showing the accumulation of 26S proteasomes in poly-GA aggregates [26]. The most enriched functional GO Biological Process terms for poly-GA associated proteins were “protein folding” and “ubiquitin protein ligase binding” (Fig. 2B). These GO terms encompass calreticulin (CALR), which was the most enriched protein in poly-GA samples when compared to myc-BioID specificity controls (Additional file 1: Supplementary Fig. 1B). Calreticulin is the ER resident chaperone that facilitates glycoprotein folding in the ER lumen and is involved in ER-associated degradation (ERAD) of misfolded proteins [37]. Importantly, another highly enriched poly-GA associated protein in this study was the autophagy receptor SQSTM1/p62, which is a well-known poly-GA interactor and robust neuropathological screening marker for DPR pathology in c9FTD/ALS cases [57, 60, 67]. Other poly-GA associated proteins from these functional categories include small heat shock proteins from the HSP70-family of chaperones HSPA1A/HSP70-1, HSPA2/HSP70-2 and HSPA8/HSC70, small heat shock protein HSPB1/HSP27, co-chaperones DNAJA1 and BAG3, as well as the key ERAD and autophagy regulator valosin-containing protein/ATPase p97 subunit (VCP/p97) and known poly-GA associated protein RAD23B/HR23B (Fig. 2C) [60, 78, 101]. Along with VCP that facilitates the degradation of aberrant proteins by segregating them from organelles or large protein complexes [97], the VCP adaptors UBXN1/SAKS1 and UBXN6/UBXD1 were also found to be associated with poly-GA. For our validation experiments we focused on how this network of molecular chaperones and co-chaperones associate with poly-GA in response to the formation of cytoplasmic poly-GA protein aggregates.

### Molecular chaperones are sequestered by poly-GA aggregates in cell culture

**Fig. 3:**
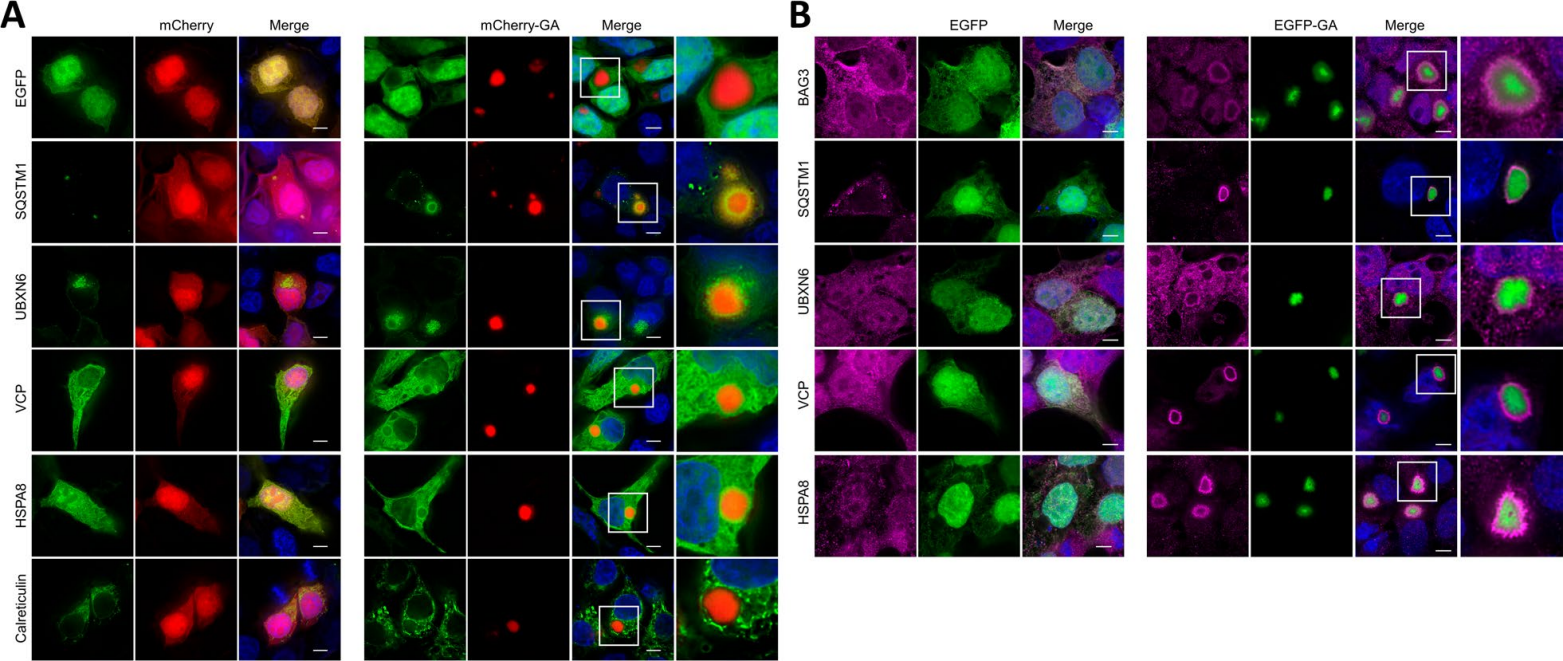
Molecular chaperones are sequestered by cytoplasmic poly-GA aggregates. **A** Co-expression of mCherry-tagged poly-GA and EGFP-tagged chaperones and adapter proteins shows the tight association of SQSTM1, UBXN6, VCP, and HSPA8 within the periphery of dense cytoplasmic poly-GA aggregates. **B** Endogenous BAG3, SQSTM1, UBXN6, VCP, and HSPA8 proteins are sequestered around EGFP-tagged poly-GA aggregates, disrupting their normal endogenous staining pattern. Scale bar: 5 µm

To better understand the relationship between poly-GA aggregates and components of cellular protein folding, quality control, and protein degradation pathways, we investigated their co-localization in cell culture using two complementary methods. In an antibody-independent approach, HEK293T cells were co-transfected with constructs for mCherry-tagged poly-GA and EGFP-tagged chaperones and co-chaperones (Fig. 3A). mCherry-poly-GA forms compact cytoplasmic aggregates, with co-localization of EGFP-tagged interactors presenting with halo-like staining that surrounds the inclusions in single optical sections, as observed from 3D deconvolved image stacks using high resolution fluorescence microscopy. Strong overlap was found for SQSTM1, while a more peripheral association was observed for co-expressed UBXN6, HSPA8 and VCP, whereas CALR showed a more distal association (Fig. 3A). In our second approach, we investigated potential sequestration of cellular proteins into poly-GA aggregates by expressing EGFP-tagged poly-GA and immunostaining fixed cells for endogenous protein candidates (Fig. 3B). High resolution fluorescence microscopy demonstrates a very similar staining pattern of endogenous proteins as compared to the EGFP-tagged fusion proteins, with BAG3, SQSTM1, UBXN6, VCP, and HSPA8 localized at the periphery of the cytoplasmic poly-GA aggregates. Their sequestration to the poly-GA inclusions and loss of normal cellular localization suggests potential defects in the normal cellular functions of these proteins (Fig. 3B). Of note, we observed a concentric staining pattern for both exogenous EGFP-tagged and endogenous interactors, with SQSTM1 staining overlapping with the periphery of the poly-GA aggregates, while HSPA8, UBXN6 and especially VCP appeared more peripheral and separated from the poly-GA aggregate. This spatial pattern is consistent with a role for UBXN6 acting as an adaptor for recruiting VCP to poly-GA aggregates that are decorated by SQSTM1, similar to its role as a VCP adaptor for aggresomes and poly-Q containing aggregates [68]. While the GFP-tag introduces lysine residues that may be directly ubiquitinated, previous studies have shown that expression of lysine-free HA-tagged poly-GA in cells leads to formation of poly-GA aggregates that are both ubiquitin- and p62-positive [96, 100]. To exclude a tag-specific artifact, we repeated co-localization experiments using expression construct encoding 2xHA-tagged poly-GA that lacks any lysine residues and found a similar association with VCP and HSPA8 (Additional file 1: Supplementary Fig. 3).

### UBXN6, HSPA8, and VCP co-localize with SQSTM1-positive poly-GA aggregates in the brain of c9FTD/ALS cases

**Fig. 4:**
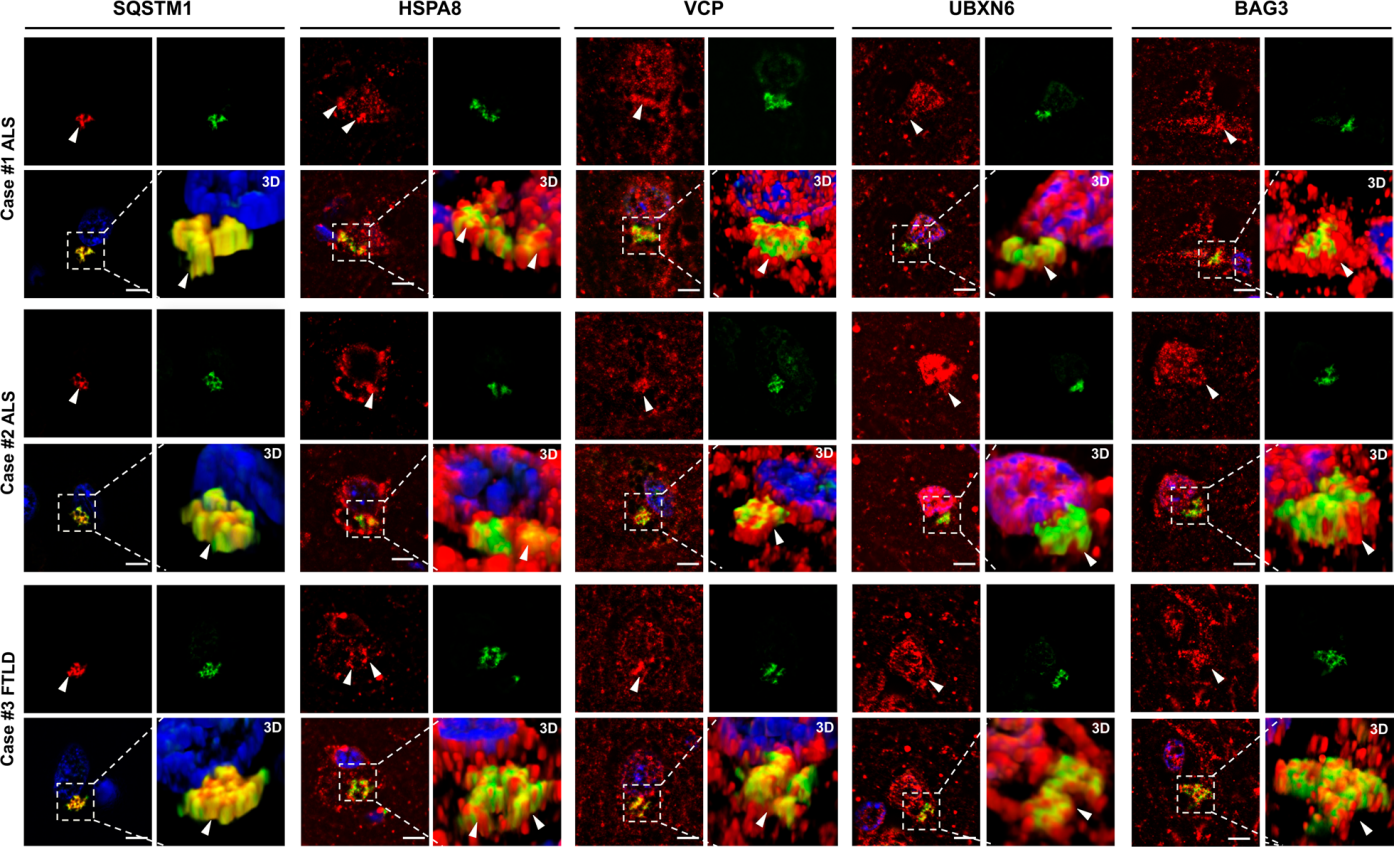
Molecular chaperones and co-chaperones closely associate with insoluble poly-GA DPR aggregates in human post-mortem brain tissue. Immunofluorescence co-staining for SQSTM1, HSPA8, VCP, UBXD1 and BAG3 (red) with poly-GA (green) and Hoechst (blue) in frontal cortex derived from neuropathologically diagnosed ALS and FTLD cases. Each staining is shown as a panel of single optical sections for red, green, and merged channels and an inset of the volume rendered z-stacks (3D). Scale bar: 5 µm

To validate our in vitro findings and establish their relevance for c9FTD/ALS pathogenesis, we determined whether these chaperones localize to poly-GA pathology in human patient brains. For this purpose, we performed stringent fluorescence double-labeling experiments in frontal cortices selected c9FTD/ALS cases with C9orf72 repeat expansion pathology from the Mayo Clinic brain bank (Table 1). High resolution fluorescence imaging was performed using a 100 × objective to acquire z-series of optical sections for deblurring and 3D image reconstructions. Great care was taken to block autofluorescence and adjust imaging parameters to prevent overexposure. Using these stringent conditions, we observed co-localization patterns that closely matched results from our cell culture model, except that poly-GA aggregates appear round and smooth in cells, probably due to the fast kinetics of overexpression and aggregation, while their shape in brain tissue was often star-shaped or peri-nuclear crescent-shaped and appeared irregular as described before [85] (Fig. 4). Similar to the close association observed in cells, SQSTM1 showed highly overlapping localization with poly-GA, visible as yellow pixels in merged images (Fig. 4), corroborating their diagnostic utility in the neuropathological screening and diagnosis of c9FTD/ALS. In addition to the optical sections, here we present volume rendered z-stacks to show the true nature of their co-localization within cells in 3D. Confirming our cell culture studies, we found that HSPA8, VCP, UBXN6 and BAG3 showed intimate co-localization and permeated cytoplasmic frontal cortex poly-GA aggregates, suggesting that they are recruited to ubiquitinated and SQSTM1-positive DPR aggregates. A similar staining pattern was observed in the temporal cortex and hippocampal dentate gyrus of c9FTD/ALS cases (Additional file 1: Supplementary Fig. 4 and 5, respectively), except that colocalization with BAG3 was not observed in all cases.

### DNAJB protein family co-chaperones reduce the formation of poly-GA aggregates

**Fig. 5:**
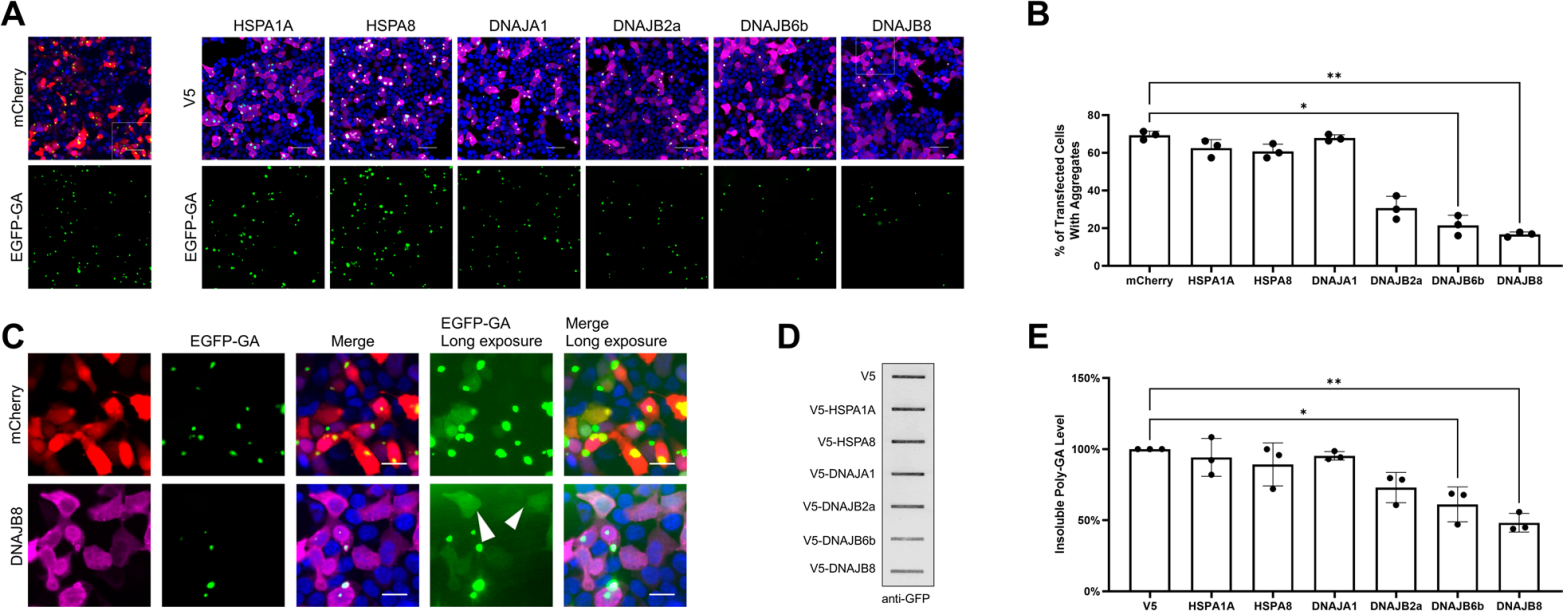
DNAJB6b and DNAJB8 co-chaperones reduce poly-GA aggregate formation and increase its solubility. **A** The EGFP-poly-GA expression plasmid was co-transfected with expression plasmids for mCherry or selected chaperones and co-chaperones. Expression of DNAJB family proteins DNAJB6b and DNAJB8 strongly reduced the formation of cytoplasmic aggregates of poly-GA and caused its diffuse nucleocytoplasmic localization. (Scale bar: 50 µm; Hoechst DNA staining in blue). **B** Quantitative image analysis shows a decrease in the percentage of cells with poly-GA aggregates when DNAJB6b and DNAJB8 are co-expressed; n = 3, mean ± SD is shown. *p < 0.05, **p < 0.01 by Kruskal–Wallis test and Dunn’s multiple comparisons post-hoc test (number of post-hoc comparisons = 6). **C** Representative images of cells co-expressing DNAJB8 vs. mCherry show a shift from bright EGFP-positive foci to diffuse EGFP staining, indicating increased solubility of EGFP-poly-GA. Scale bar: 20 µm. **D** Filter trap assay (FTA, representative example) and **E** FTA quantification of poly-GA aggregates shows a reduction in detergent-insoluble poly-GA aggregates in lysates from cells co-transfected with DNAJB2a, DNAJB6b and DNAJB8; n = 3, mean ± SD is shown. *p < 0.05, **p < 0.01 by Kruskal–Wallis test and Dunn’s multiple comparisons post-hoc test (number of post-hoc comparisons = 6)

HSP40/DNAJ family proteins acting as co-chaperones for HSC/HSP-70 ATPases have been shown to protect against neurodegeneration caused by misfolding and aggregation of various proteins, including poly-Q expanded proteins, amyloid-β, α-synuclein, tau, Parkin, SOD1, and TDP-43 [99]. In our proximity proteomics data set we identified DNAJA1 as a novel poly-GA associated protein (Fig. 2C). This largest chaperone family consists of the DNAJA, DNAJB, and DNAJC sub-families with different domain structures. Since its members are characterized by tissue-specific expression patterns including the CNS, they may not be active in HEK293T cells [99]. In a previous pilot screen of DNAJ family protein for reduction of poly-GA aggregation, we had identified several DNAJB protein family members as potential modifiers of poly-GA aggregate formation (data not shown). Of note, co-chaperones DNAJA1 and DNAJB6 have previously been identified as modulators of poly-Q huntingtin (htt) aggregation in a cellular Huntington’s disease model, with the knock-out (KO) of DNAJB6 resulting in a fivefold increase in poly-Q74htt aggregation while a DNAJA1 KO resulted in a fourfold decrease of poly-Q74htt aggregation [79]. DNAJB6 is most closely related to its protein family members DNAJB2 and DNAJB8, which share a broad activity towards different neuropathological aggregates [99]. Both DNAJB6 and DNAJB8 have been shown to prevent the aggregation of pure polyQ peptides in vitro [25]. To investigate the effect of these HSP40/DNAJ family proteins on poly-GA aggregation, we co-transfected HEK293T cells with expression plasmids for EGFP-tagged poly-GA and V5-tagged co-chaperones. We selected candidates for our assays based on our BioID dataset and added DNAJB family proteins identified in a previous screen as described above. The complementary use of imaging and biochemical assays allowed us to determine and quantify their effect on poly-GA aggregation and solubility. In the microscopy-based assay, we assessed the presence of EGFP-poly-GA foci vs. soluble protein GA in co-transfected cells. Filter trap assays were used to measure detergent-solubility of poly-GA in the resulting lysates. Expression of the co-chaperones DNAJB6b and DNAJB8 strongly reduced the formation of poly-GA aggregates, whereas DNAJA1 and the chaperones HSPA1A and HSPA8 had no significant effect (Fig. 5A and B). We also observed that DNAJB2a could visibly reduce poly-GA aggregation, although the difference between DNAJB2a and mCherry control conditions did not reach statistical significance (Fig. 5A, B, D and E). HSPA8 showed the strongest co-localization with poly-GA aggregates, while a weaker association is observed for the co-chaperones from the HSPB family. Overexposing images reveal the presence of diffuse EGFP-poly-GA distribution in cells expressing active DNAJB co-chaperones to a much greater extent than in controls, where EGFP-poly-GA predominantly forms round inclusions due to the high propensity of poly-GA to aggregate (Fig. 5C). In the filter trap assay, we assessed the detergent solubility of poly-GA protein when co-expressed with molecular chaperones. Confirming the results from microscopy, we found a decrease in 2% SDS-insoluble poly-GA aggregates when DNAJB6b and DNAJB8 were co-expressed (Fig. 5D and E). Taken together, our results establish that the same group of co-chaperones from the DNAJB family that reduce poly-Q, α-synuclein, and TDP-43 aggregation [1, 2, 10, 25], also strongly reduce the formation of insoluble poly-GA aggregates. These findings suggest that current efforts to harness the activity of cellular chaperone networks to target neuropathological aggregates may be used to target poly-GA aggregates in c9FTD/ALS patients [17, 88, 99].

## Discussion

In this study we report that proximity-proteomics is a highly valuable strategy for interrogating the composition and associated proteome of neuropathological inclusions, which allowed us to identify key factors that could be exploited as therapeutic modifiers of DPR pathogenesis in c9FTD/ALS. While most interactome studies employ classical immunoaffinity purification, we have found that proximity-dependent biotin identification (BioID) can be used to profile the composition of detergent-insoluble aggregates, which has led to our previous discovery of nucleocytoplasmic transport defects associated with TDP-43 pathology [12]. Unlike conventional co-immunoprecipitation approaches in cell lysates, BioID and related methods using biotin ligase variants (e.g. TurboID) or engineered peroxidases (APEX2) allow for spatiotemporal labeling the components and closely associated proteins of insoluble aggregates in the context of living cells, followed by harsh solubilization conditions (e.g. 8 M urea) and high-affinity biotin-streptavidin purification for mass spectrometry based proteomics [82]. APEX2 is more suitable for capturing the composition of dynamic condensates and has been used for the spatiotemporal characterization of the stress granule proteome, and for evaluating the effect of poly-PR on the stress granule proteome [59]. For poly-GA that forms compact and highly insoluble aggregates composed of amyloid fibrils, conventional affinity purification methods have yielded fewer interactors than for charged arginine-rich poly-GR and -PR [49]. Although the dense nature of myc-BioID-poly-GA aggregates appeared to limit the penetration of anti-myc antibodies, it does not impede proximity labeling with small biotin molecules in living cells, allowing us to identify numerous and novel poly-GA interactors (Fig. 1A).

Our proximity proteomics data for poly-GR and -PR are consistent with the literature, showing enrichment for nucleolar and ribosomal proteins [32]. Expression of these arginine-rich repeats in cell culture shows a stronger staining of nucleoli than has been reported from human brain tissue, whereas the subcellular distribution of poly-GA aggregates matches human neuropathological findings [85]. While we also discovered novel poly-GR/PR interactors with potential links to c9FTD/ALS pathophysiology as described above and in Fig. 2, in our validation experiments and functional assays we focused on the poly-GA interactome. The highly aggregation prone poly-GA can induce significant neurotoxicity in cellular and animal models of c9FTD/ALS [26, 40, 41, 47, 60, 72, 74, 84]. While poly-GR can recruit TDP-43 into cytoplasmic inclusions [14], poly-GA may promote TDP-43 aggregation more indirectly via inhibition of proteasome mediated protein degradation [41, 73]. Studies in mouse models report poly-GA toxicity causing neuroinflammation and additional defects caused by the sequestration of proteins into insoluble aggregates [41, 47, 84, 100, 101]. For these reasons, interrogating poly-GA interactomes and affected pathways will contribute to a better understanding of c9FTD/ALS relevant disease processes and therapeutic targets [33].

Our validation experiments included overexpression of tagged proteins and immunocytochemistry of endogenous proteins in cell culture (Fig. 3A and B) and immunohistochemistry in patient-derived brain tissue from neuropathologically confirmed c9ALS and c9FTLD autopsy cases. Using high resolution fluorescence microscopy with deconvolved image stacks, we found a very consistent pattern across these experiments, with SQSTM1 showing a very close association and co-localization with poly-GA aggregates, while VCP, its adaptor protein UBXN6 and the chaperones HSPA8 and BAG3 demonstrated close association and permeation of poly-GA inclusion, but not completely overlapping co-localization. These findings not only validate our proximity proteomic interactome results but also suggest a distinct spatial relationship with SQSTM1 decorating ubiquitinated aggregates, while chaperones appear to be sequestered within and towards the periphery of the inclusions. These findings also highlight a specific feature of proximity labeling vs. co-immunoprecipitation of physically associated proteins that allows us to interrogate the molecular environment of protein complexes. While the labeling radius for BioID in endogenous protein complexes has been estimated at approximately 10 nm [42], the use of an flexible linker in the fusion proteins and its high local concentration in aggregates may expand labeling to some extent [43].

The poly-GA interactome was highly enriched for proteins involved in protein folding, ubiquitination, and degradation. STRING pathway analysis showed one major node group for proteasome components, consistent with the accumulation of cellular proteasomes in poly-GA aggregates [26]. Another major group of nodes in the interactome network is clustered around the ATPase VCP, and its adapter proteins UBXN1 and UBXN6. This cluster of functionally connected interactors also encompasses the Ub-associated (UBA) domain ubiquitin receptor for proteasomal degradation RAD23B, and the autophagic ubiquitin receptor SQSTM1 [105]. In a murine model of poly-GA pathology and in human *C9orf72* expansion carriers RAD23B was found to be sequestered into poly-GA inclusions, while SQTSM1 is a known reliable marker for DPR pathology in c9FTLD/ALS [58, 101]. Another member of this group is the ER chaperone CALR, which has multiple functions including the regulation of Ca2^+^ homeostasis and protein folding, and was found dysregulated in neurological disorders, including ALS [45]. Of note, a study of motor dysfunction in a poly-GA mouse model has shown mobile poly-GA aggregates within neurites, causing altered Ca2^+^ influx and synaptic vesicle release [36]. It would be interesting to see whether these defects are mediated via the association of poly-GA with CALR.

Dysfunction of autophagy-related proteins impairs proteostasis and causes neurotoxicity in FTD/ALS, and mutations in both SQSTM1 and VCP can cause ALS and FTD [13]. VCP, in combination with adaptor proteins such as UBXN1 and UBXN6, functions to identify misfolded proteins, extract them from aggregates and damaged mitochondria and lysosomes, and target them for degradation by the proteasome or through autophagy [22, 27, 75]. VCP and its adaptor FAF2 were recently found to mediate the extraction of G3BP1 from stress granules induced by heat stress, leading to their disassembly [29], which establishes an interesting connection between stress granule dynamics and the pathogenesis of proteinopathies [52]. SQSTM1 serves as an autophagy receptor protein that binds both LC3-II and polyubiquitinated proteins, targeting ubiquitinated substrates to autophagosomes for degradation [13]. Taken together, these findings suggest a direct link of poly-GA aggregates to autophagy as a substrate but may also reflect a dysregulation of both major pathways of protein degradation caused by the sequestration of autophagy receptors and proteasomal subunits.

These autophagy-related proteins are functionally connected to another cluster of proteins containing the heat shock proteins HSPA8, HSPA1A, HSPB1, SERPIN H1/HSP47, the co-chaperones BAG3 and DNAJA1, and the E3 ubiquitin ligase CHIP, which targets misfolded chaperone substrates towards proteasomal degradation. Mutations in BAG3, HSPB1, and CHIP have been linked to neurodegenerative diseases [83]. In addition, we also found ubiquitin C-terminal hydrolase L1 (UCHL1) and Parkinson disease protein 7 (PARK7/DJ-1) that are closely associated with the above-mentioned molecular chaperones in the STRING interaction network and are both linked to Parkinson’s disease [7, 51].

Supporting evidence for a disease-relevance of our findings comes from a transcriptomics study that found an induction of a heat shock response caused by C9orf72 pathology in c9FTL/ALS brain tissue relative to both sporadic cases and controls [65]. The upregulated network of transcripts under control of the HSF1 transcription factor includes several poly-GA associated proteins that we have identified in this study, such as BAG3, HSPB1/HSP27, SERPIN H1/HSP47, HSPA1A, but also DNAJB proteins. This study also found a significant upregulation of HSPA1B transcripts in human neurons treated with poly-GA and poly-GR, supporting a role for DPR-specific gain-of-function effects in activating a heat shock response [65].

HSP70 family chaperones play a key role in ATPase mediated correct folding of aberrant protein substrates under normal conditions but also under cellular stress exposure and in neurodegenerative proteinopathies, which has led to development of therapeutic strategies targeting their upregulation [28]. HSP70/HSC70 proteins are also regulated by their association with co-chaperones and other factors. Notably, the ALS/FTD risk factor UBQLN2 can recognize ubiquitinated HSP70 to facilitate the clearance of poly-GA aggregates, thereby alleviating neurotoxicity in a poly-GA animal model [100]. Here we found an association with the C-terminal HSC70-interacting protein (CHIP), which acts as a chaperone dependent E3 ligase that ubiquitinates unfolded proteins and can bind to HSC70 and HSP70, and can switch chaperone activity from protein folding to protein degradation [20]. Another regulatory co-chaperone identified here is BAG3, which is implicated in rerouting UPS substrates to BAG3-mediated selective macroautophagy. BAG3 in concert with HSP70/HSC70 as well as the ubiquitin receptor p62/SQSTM1 specifically targets aggregation-prone proteins to autophagic degradation under conditions of cellular stress and in the context of aging and neurodegenerative diseases [89]. We also identified DNAJA1, which belongs to a large family of DNAJ/HSP40 co-chaperones that target misfolded target protein substrates to specific HSP70 chaperones for refolding. Their tissue-specific expression, high substrate specificity, and potent activity towards targeting misfolded disease proteins makes them an attractive target for therapeutic interventions of neurodegenerative disorders [4]. Our identification of this poly-GA associated network of functionally related HSP70/HSC70 chaperones and co-chaperones suggest its critical role in protein aggregate clearance in c9FTD/ALS.

This work also reveals a novel role of the DNAJB family of co-chaperones that have been previously shown to reduce poly-Q and α-synuclein aggregation [1, 19, 25, 79] and modulate their pathology in animal models of Parkinson’s disease and Huntington’s disease [2, 38], in strongly reducing the formation of insoluble poly-GA aggregates (Fig. 5A and B). This subfamily of DNAJB proteins comprises the closely related DNAJB2a, DNAJB6b and DNAJB8 proteins, which were the most efficient at reducing poly-Q aggregation [31]. While these proteins were not identified in the proximity proteome in HEK293T cells, they are normally expressed in a tissue specific manner and we selected them for further investigation due to their activity towards poly-GA aggregation in a screen and their well-documented activity towards neuropathological inclusions in the literature [99]. Recessive mutations in the gene encoding the neuronal DNAJB2/HSJ1 protein can result in distal hereditary motor neuronopathy, Charcot-Marie-Tooth disease type 2, or spinal muscular atrophy/juvenile Parkinsonism [99]. DNAJB6/MRJ is ubiquitously expressed but most abundantly localized within the brain and in muscle tissue, with mutations causing dominant limb-girdle muscular dystrophy [99]. In patients carrying DNAJB6 mutations this anti-aggregation property is reduced, and they present with myofibrillar inclusions positive for ubiquitin, SQSTM1 and TDP-43, suggesting defective protein clearance [81]. Our results suggest that therapeutic efforts to target the activity of DNAJB6 and related co-chaperones in synucleinopathies and poly-Q disorders may also be beneficial for c9FTD/ALS patients, potentially targeting both TDP-43 and poly-GA aggregation. It remains to be observed whether the broad target specificity of these DNAJB family co-chaperones results from recognizing common structural features on the misfolded protein substrates or shared interactors of these divergent neuropathological aggregates [4].

While this manuscript was in preparation, a related study based on a similar strategy was published [8]. Although some of our results overlap, including the identification of VCP in the poly-GA interactome, we also identified numerous additional poly-GA associated proteins, including VCP adapters, molecular chaperones and co-chaperones, and SQTSM1, a highly reliable marker for poly-GA pathology in vitro and in autopsy tissue. This difference may be due to our method that we have optimized for the study of neuropathological inclusions, such as using more stringent lysis conditions in our study (8 M urea instead of 0.2% SDS + 2% Triton X-100) for solubilizing detergent-insoluble poly-GA aggregates and associated proteins, which allows us to also identify insoluble co-depositing proteins involved in c9FTD/ALS pathogenesis.

Of note, therapeutic strategies targeting poly-GA have shown promising results in C9orf72 mouse models. Active poly-GA vaccination by immunizing a C9orf72 mouse model with a poly-GA fusion protein markedly reduced neuroinflammation, motor deficits, cytoplasmic TDP-43 mislocalization and CSF levels of neurofilament light chain, the latter of which is a reliable biomarker of neurodegeneration [103]. Passive immunotherapy based on injecting C9orf72-BAC mice with a human anti-GA antibody reduced not only poly-GA, but also -GP, and -GR aggregates, which subsequently improved behavior and survival, whilst also decreasing neurodegenerative read-outs [71]. Taken together, these data suggest that immunotherapies or other strategies primarily targeting poly-GA pathology may be a suitably viable therapeutic approach for c9FTD/ALS [33]. Neuropathological and biomarker studies suggest that targeting early prodromal DPR pathology may prevent the later onset of symptoms and the appearance of TDP-43 pathology and resultant neurodegeneration [5, 23, 50, 93].

While this work and other studies provide evidence for poly-GA aggregates causing defects in protein degradation, it remains unclear how poly-GA pathology triggers neuroinflammation in mouse models and potentially c9FTD/ALS in human brains [47, 71, 84, 103]. Reduced C9orf72 protein levels [104], the presence of RNA repeat pathology, and the co-aggregation of multiple DPRs as separate molecules or chimeric peptides [61] are expected to contribute to the complexity of DPR pathologies and their spectrum of clinicopathologic presentations. Future studies in vertebrate animal models that combine multiple aspects of C9orf72 neuropathology, neuroinflammation and neurodegeneration and studies utilizing the rich resource of diagnostically confirmed human brain tissue will be required to obtain a more complete picture of the timeline and specific contributions of DPR pathologies in the disease process.

## Supplementary Information

**Additional file 1.** Supplementary Fig. 1, 2, 3, 4, 5.

**Additional file 2.** Supplementary Table 1.

## Data Availability

Raw proteomic data files along with the Spectronaut search results, quantification and statistical analysis of proteomic data were submitted to ProteomeXchange Consortium via MassIVE mass spectrometry data repository (MassIVE identifier MSV000088581, ProteomeXchange identifier PXD030490).
