# Supplementary material for: Proximity proteomics of C9orf72 dipeptide repeat proteins identifies molecular chaperones as modifiers of poly-GA aggregation
Source: Acta Neuropathol Commun. 2022 Feb 14;10:22. doi: 10.1186/s40478-022-01322-x (PMC8842533; doi:10.1186/s40478-022-01322-x)
Supplement: Supplementary file 1 — Additional file 1. Supplementary Fig. 1, 2, 3, 4, 5. [file 40478_2022_1322_MOESM1_ESM.pdf]

Acta Neuropathologica Communications

Proximity proteomics of C9orf72 dipeptide repeat proteins identifies molecular chaperones as modifiers of poly-GA aggregation

Feilin Liu[1,2], Dmytro Morderer[1], Melissa C. Wren[1], Sara Vettleson-Trutza[1], Yanzhe Wang[1,3], Benjamin E. Rabichow[1], Michelle Salemi[4], Brett S. Phinney[4], Björn Oskarsson[5], Dennis W. Dickson[1], and Wilfried Rossoll[1]

[1] Department of Neuroscience, Mayo Clinic, Jacksonville, FL, USA

- Correspondence: rossoll.wilfried@mayo.edu

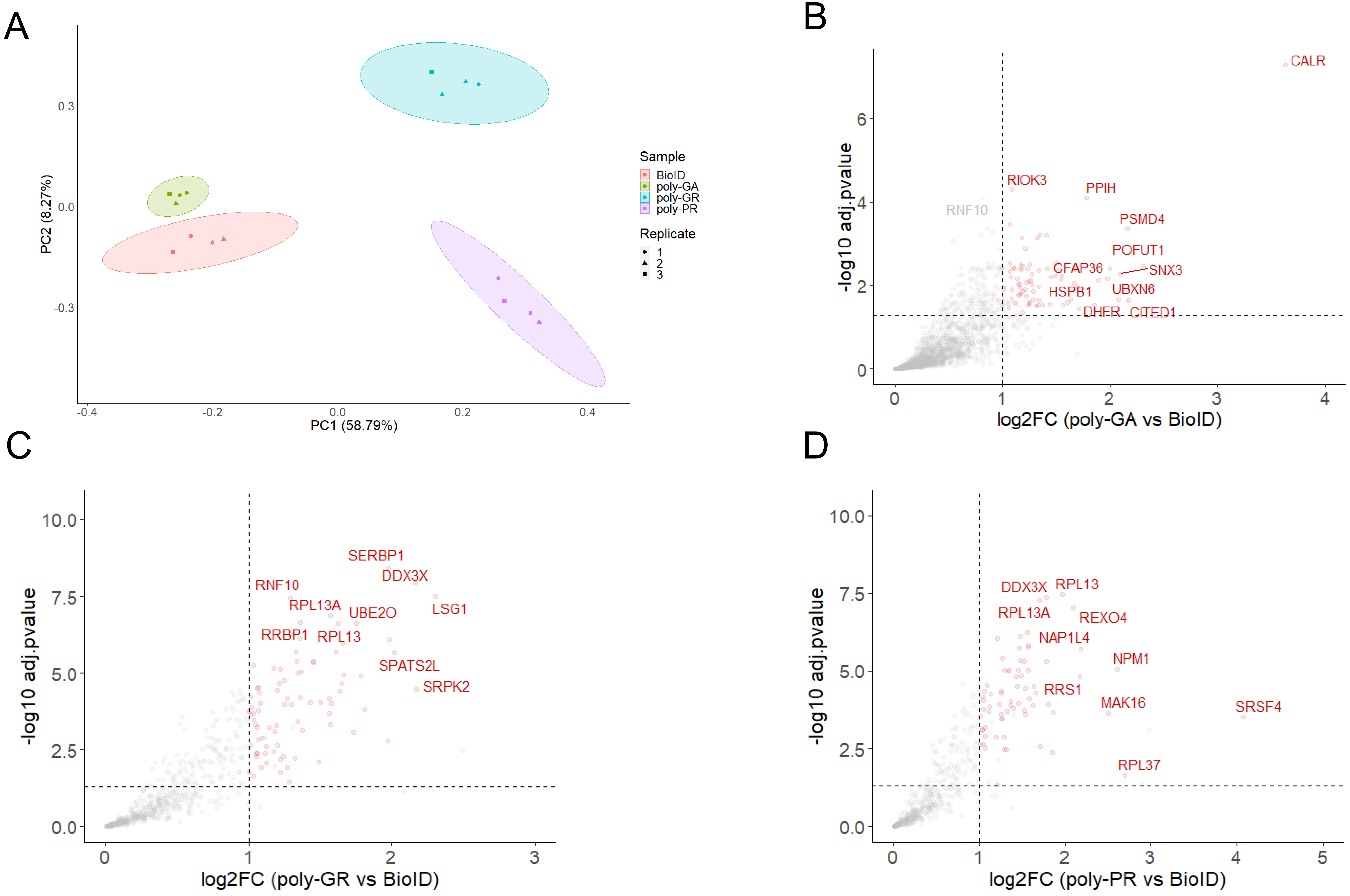

Supplementary Fig. 1. A) Principal component analysis plot for interactomes of samples used in this study (n=4); **B-D**) Volcano plots for the comparisons of protein abundances in poly-GA (**B**), poly-GR (**C**) and poly-PR (**D**) samples with the myc-BioID specificity control samples.

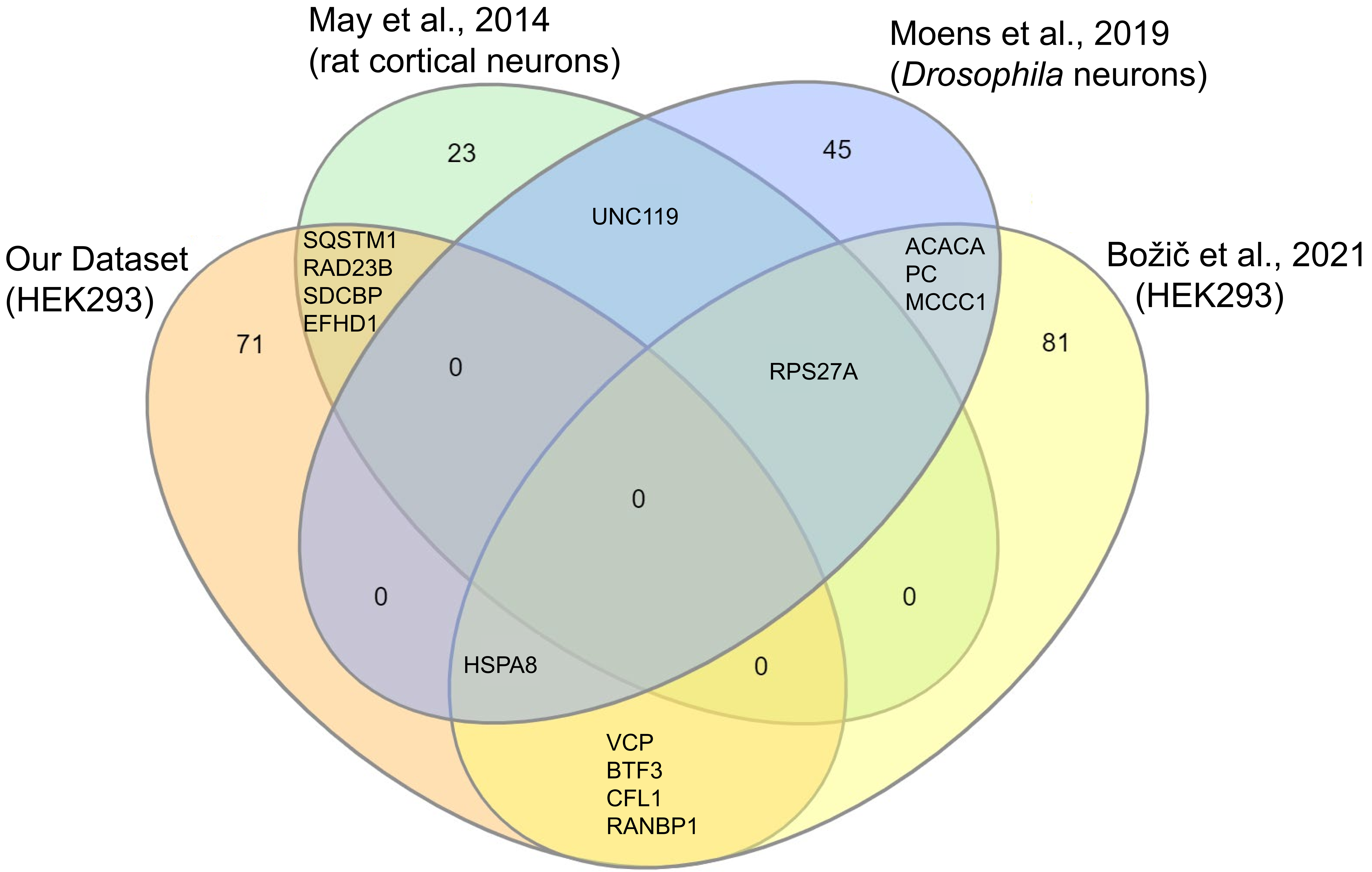

Supplementary Fig. 2. Comparison of the poly-GA associated protein dataset from this study with previously published datasets.

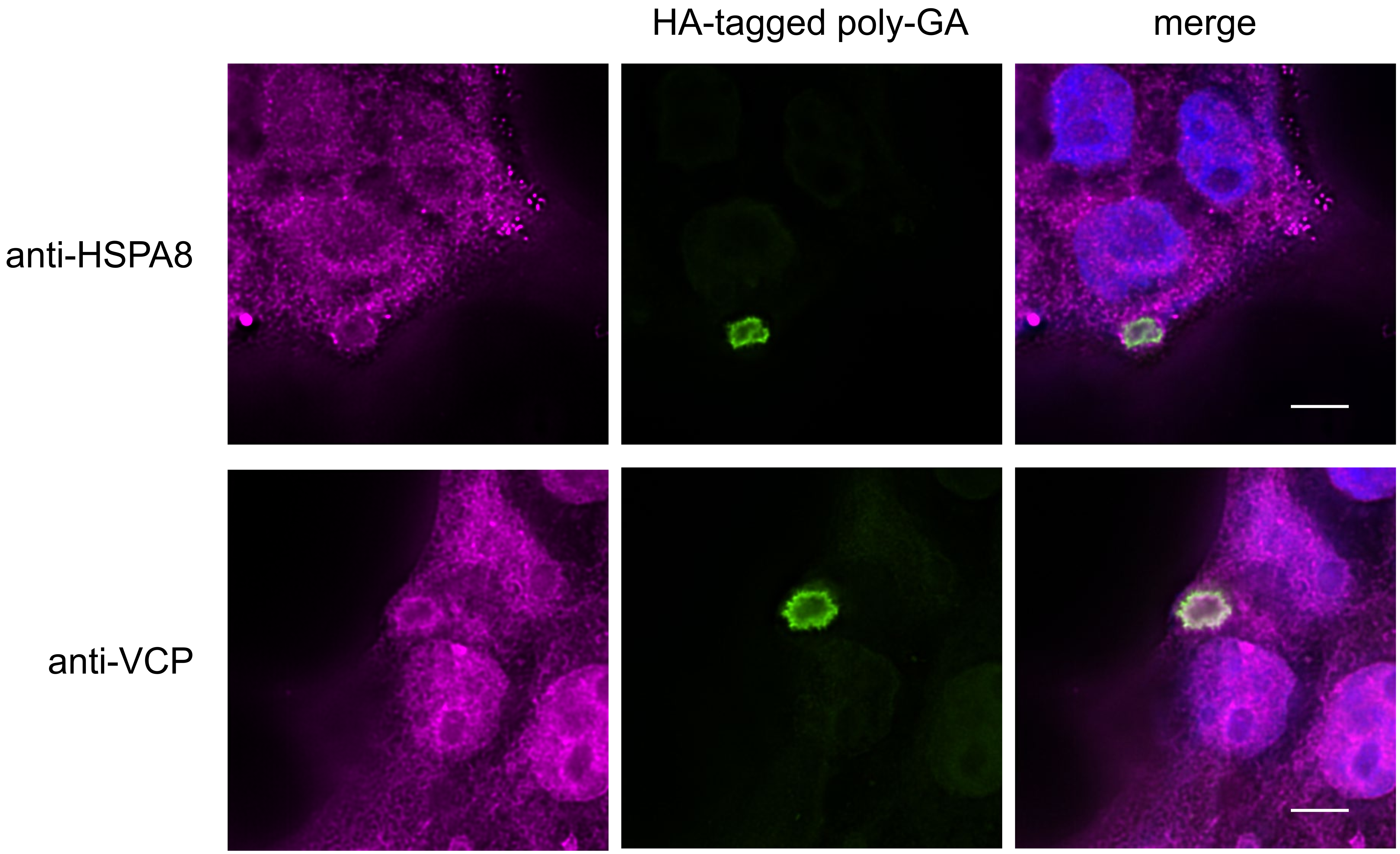

Supplementary Fig. 3. Molecular chaperones are sequestered by cytoplasmic poly-GA aggregates lacking lysine residues. Endogenous HSPA8 and VCP proteins are sequestered around HA-tagged poly-GA aggregates. Size bar = 5μm.

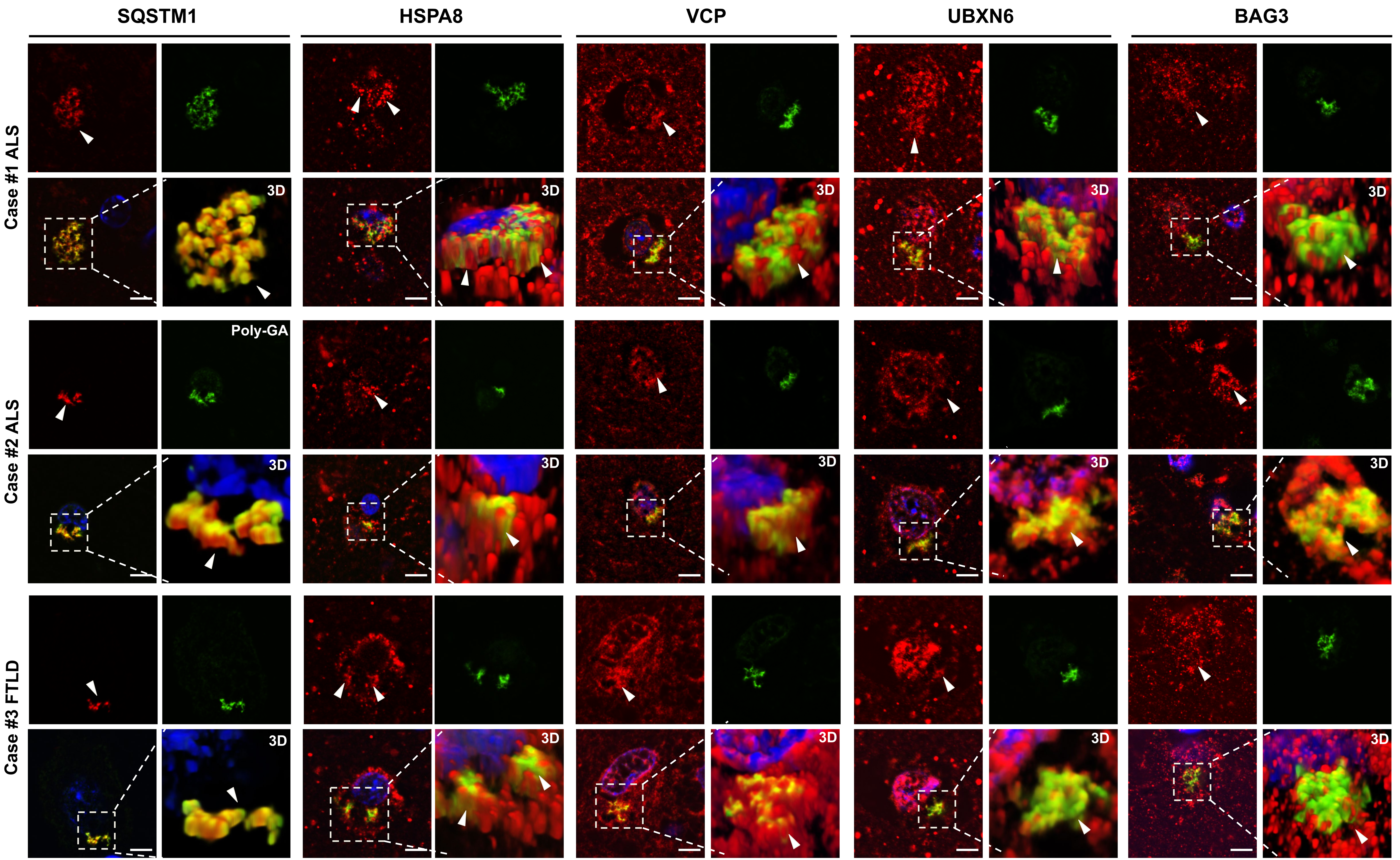

Supplementary Fig. 4. Molecular chaperones and co-chaperone association with insoluble poly-GA DPR aggregates in temporal cortex post-mortem tissue. Immunofluorescence co-staining for SQSTM1, HSPA8, VCP, UBXD1 and BAG3 (red) with poly-GA (green) and Hoechst (blue) in the temporal cortex from neuropathologically diagnosed ALS and FTLD cases. Each staining is shown as a panel of single optical sections for red, green, and merged channels and an inset of the volume rendered z-stacks (3D). Scale bar: 5 µm. Abbreviations: ALS, amyotrophic lateral sclerosis; FTLD, frontotemporal lobar degeneration; DPR, dipeptide repeat protein; SQSTM1, sequestosome 1; HSPA8, heat shock protein family A member 8; VCP, valosin-containing protein; UBXN6, UBX-domain containing protein 6; BAG3, Bcl2-associated athanogene 3.

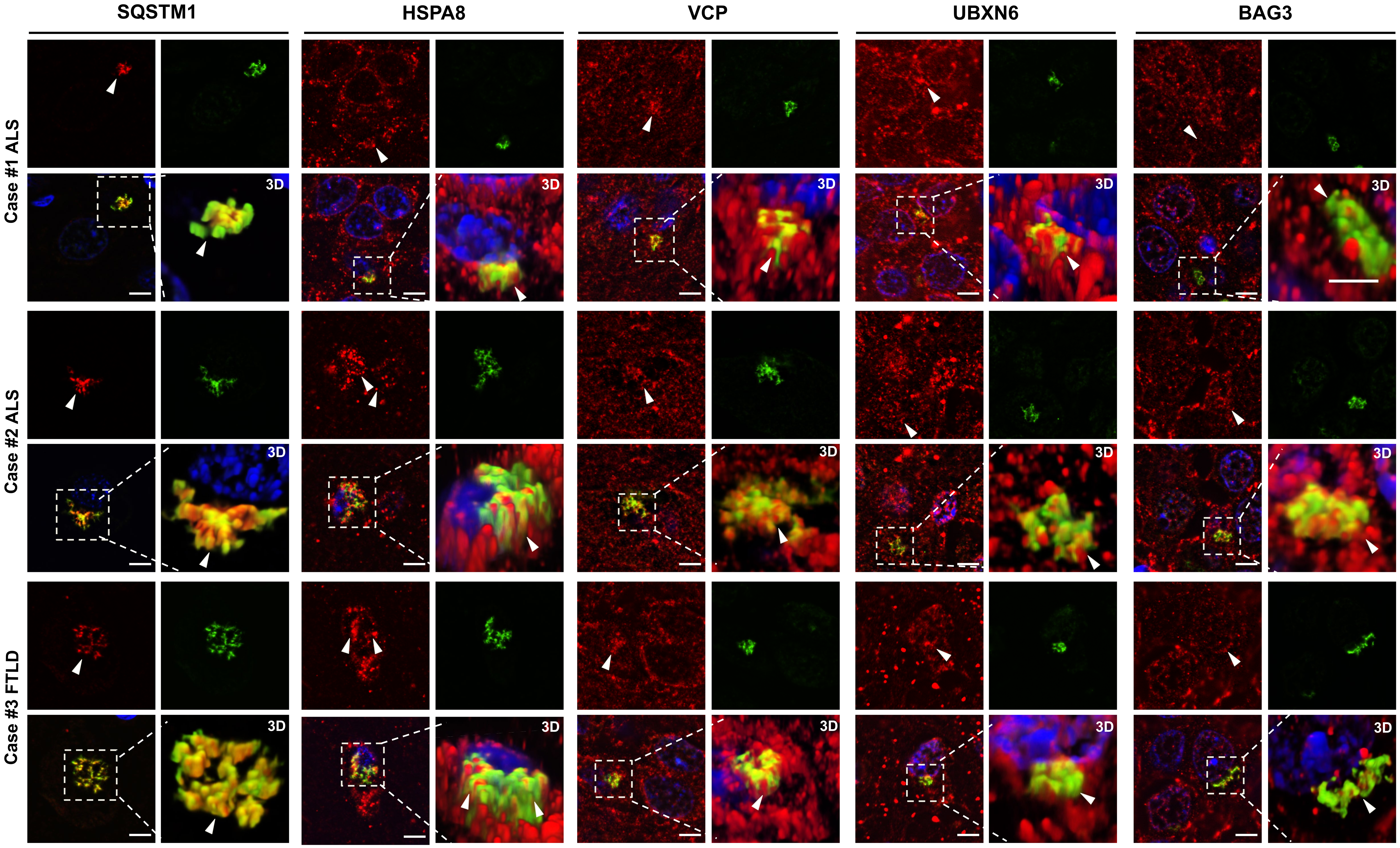

Supplementary Fig. 5. Molecular chaperones and co-chaperone association with insoluble poly-GA DPR aggregates in hippocampal human post-mortem tissue. Immunofluorescence co-staining for SQSTM1, HSPA8, VCP, UBXD1 and BAG3 (red) with poly-GA (green) and Hoechst (blue) in the hippocampus from neuropathologically diagnosed ALS and FTLD cases. Each staining is shown as a panel of single optical sections for red, green, and merged channels and an inset of the volume rendered z-stacks (3D). Scale bar: 5 µm. Abbreviations: ALS, amyotrophic lateral sclerosis; FTLD, frontotemporal lobar degeneration; DPR, dipeptide repeat protein; SQSTM1, sequestosome 1; HSPA8, heat shock protein family A member 8; VCP, valosin-containing protein; UBXN6, UBX-domain containing protein 6; BAG3, Bcl2-associated athanogene 3.
